# Microglial MAC1 receptor and PI3K are essential in mediating β-amyloid peptide-induced microglial activation and subsequent neurotoxicity

**DOI:** 10.1186/1742-2094-8-3

**Published:** 2011-01-13

**Authors:** Dan Zhang, Xiaoming Hu, Li Qian, Shih-Heng Chen, Hui Zhou, Belinda Wilson, David S Miller, Jau-Shyong Hong

**Affiliations:** 1 Laboratory of Pharmacology, National Institute of Environmental Health Sciences, National Institutes of Health, Research Triangle Park, NC 27709, USA; 2 Institute of Materia Medica, Chinese Academy of Medical Sciences & Peking Union Medical College, Beijing, 100050, PR China; 3 Department of Neurology and Pittsburgh Institute of Neurodegenerative Diseases, University of Pittsburgh School of Medicine, Pittsburgh, PA 15261, USA; 4 Comprehensive Center for Inflammatory Disorders, University of North Carolina, Chapel Hill, NC 27599, USA; 5 College of Medicine, Institute of Behavioral Medicine, National Cheng-Kung University, Tainan, Taiwan

## Abstract

**Background:**

β-Amyloid peptide (Aβ) is a major protein in the brain associated with Alzheimer's and Parkinson's diseases. The purpose of this study was to investigate the role of macrophage antigen-1 (MAC1) receptor, an integrin scavenger receptor in microglia, and subsequent signaling events in mediating Aβ-induced neurotoxicity. We have previously reported that NADPH oxidase (PHOX) on microglia and superoxide produced by PHOX are critical for Aβ-induced loss of dopaminergic neurons. However, the upstream signaling pathway of superoxide production remains unclear.

**Methods:**

For the *in vitro *study, mesencephalic neuron-glia cultures and microglia-enriched cultures from mice deficient in the MAC1 receptor (MAC1^-/-^) and wild type controls were used to investigate the role of MAC1 receptor in Aβ-induced neurotoxicity and the role of phosphoinositide-3 kinase (PI3K) in the signal pathway between MAC1 receptor and PHOX. For the *in vivo *study, Aβ was injected into the substantia nigra of MAC1^-/- ^mice and wild type mice to confirm the role of MAC1 receptor.

**Results:**

We found that Aβ-induced activation of microglia, activation of PHOX, generation of superoxide and other reactive oxygen species, and loss of dopaminergic neurons were decreased in MAC1^-/- ^cultures compared to MAC1^+/+ ^cultures. In MAC1^-/- ^mice, dopaminergic neuron loss in response to Aβ injection into the substantia nigra was reduced relative to MAC1^+/+ ^mice. Thus, MAC1 receptor-mediated PHOX activation and increased superoxide production are associated with Aβ-induced neurotoxicity. PI3K activation was one downstream step in MAC1 signaling to PHOX and played an important role in Aβ-induced neurotoxicity. In microglia-enriched cultures from MAC1^-/- ^mice, Aβ-induced activation of PI3K (phosphorylation of target proteins and PIP_3 _production) was reduced relative to MAC1^+/+ ^cultures.

**Conclusions:**

Taken together, our data demonstrate that Aβ activates MAC1 receptor to increase the activity of PI3K, which in turn phosphorylates p47^*phox*^, triggers the translocation of cytosolic subunits of PHOX to microglia membrane, increases PHOX activation and the subsequent production of superoxide and causes neurotoxicity.

## Background

β-Amyloid peptide (Aβ) has been reported to exist in numerous diseases such as Alzheimer's disease (AD), Parkinson's disease (PD), age-related macular degeneration and Lewy body disease with dementia [1-3]. Immunohistochemical analysis reveals that Aβ deposits in the brain are surrounded by and infiltrated by activated microglia [4,5], suggesting that microglia are involved in the pathogenesis of amyloid-related diseases.

The major purpose of this paper was to study the molecular signaling events mediating the neurotoxicity of Aβ. The essential role of microglia in mediating Aβ-elicited neurotoxicity was extensively compared in both cortical and midbrain cultures in our previously published paper and the results showed that they were qualitatively similar [6]. This similarity in Aβ-elicited toxicity in both cortical vs. midbrain cultures was further demonstrated in the present study. Thus, we believe that the signaling pathway mediating Aβ-elicited neurotoxicity obtained from midbrain cultures should be applicable to the cortical cultures. The main reason for using midbrain dopamine neuron-microglia cultures for the mechanistic studies in this paper was due to its unique features: 1) the high density of microglia (about 5 times more than cortical cultures) and 2) higher sensitivity of dopamine neurons to oxidative damage in the midbrain cultures. For these reasons, midbrain culture has been widely used for studying the role of microglia in oxidative insult-related neurotoxicity. For the same reason, we selected substantia nigra (SN) in the *in vivo *study so that we could quantitatively measure specific damage of dopamine neurons in a much precise and reproducible manner.

The present study is concerned with how Aβ signaling leads to the activation of NADPH oxidase (PHOX). We tested the hypothesis that signaling is mediated by macrophage antigen complex-1 (MAC1) receptor. MAC1 (integrin CD11b/CD18 receptor) is both an adhesion molecule and a pattern recognition receptor that has long been recognized to regulate diverse functions involved in adhesion, migration, phagocytosis and chemotaxis [7,8]. MAC1 was previously identified as a TLR4-independent receptor for lipopolysaccharide (LPS) in phagocytes [9]. MAC1 is elevated in the brains of post mortem AD patients [10] and in the 1-methyl-4-phenyl-1,2,3,6-tetrahydropyridine (MPTP) animal model of PD [11], suggesting a role for MAC1 in neurodegeneration. MAC1 receptor is essential for phagocytosis of multiple compounds and the activation of phagocytes in response to a diverse set of stimuli [12,13]. MAC1 is also associated with respiratory burst in neutrophils [8], suggesting that it may be a potential source of microglia-derived oxidative stress. It has also been reported that MAC1 plays a role in PHOX activation in response to oxidative insults [8,12,14]. Other evidence suggests that MAC1 occupancy is capable of triggering the transcription factor nuclear factor-κB (NF-κB) signaling pathway and the subsequent production of inflammatory factors [15,16]. There is increasing evidence that pattern recognition receptors (i.e. TLR4, scavenger receptors, and MAC1) associated with phagocytes are critical in mediating neurotoxic effects. Recently, we reported that microglial MAC1 receptor plays a critical role in mediating neurotoxicity induced by LPS, α-synuclein and MPTP [17-19].

Here, we implicate the MAC1 receptor in Aβ-induced microglial activation, and Aβ-induced neurotoxicity, *in vitro *and *in vivo*. We show that MAC1 signals through Phosphoinositide-3 kinase (PI3K) to activate PHOX-mediated superoxide generation and neurotoxicity.

## Methods

### Reagents

Aβ(1-42) was purchased from the American Peptide Company (Sunnyvale, CA, USA). WST-1 was purchased from Dojindo Laboratories (Gaithersburg, MD). Cell culture ingredients were obtained from Invitrogen (Carlsbad, CA). [^3^H] dopamine (30 Ci/mmol) and [2,3-^3^H] γ-Aminobutyric acid (GABA) (81 Ci/mmol) was purchased from Perkin Elmer Life Sciences (Boston, MA). The polyclonal antibody against tyrosine hydroxylase (TH) was a kind gift from Dr. John Reinhard of GlaxoSmithKline (Research Triangle Park, NC). Monoclonal antibody against the F4/80 was obtained from Serotec (Raleigh, NC). Polyclonal antibody against Iba1 was obtained from Wako Chemicals USA, Inc. (Richmond, VA). Vectastain avidin-biotinylated enzyme complex, biotinylated horse anti-mouse and goat anti-rabbit secondary antibodies were purchased from Vector Laboratories (Burlingame, CA). Rabbit anti-p47^*phox *^antibody was obtained from Upstate (Lake Placid, NY). FITC-conjugated goat anti-rabbit IgG antibody was obtained from Jackson ImmunoResearch Laboratories (West Grove, PA). Rabbit anti-GAPDH, rabbit anti-AKT (phosphor S473) antibodies were obtained from Abcam (Cambridge, MA). Mouse anti-gp91^*phox *^antibody was purchased from BD Transduction Laboratories (San Jose, CA). Rabbit anti-PI3K p110 antibody was purchased from Santa Cruz Biotechnology, Inc. FITC-conjugated anti phosphoinositide(3,4,5)bisphosphate (PIP_3_) antibody was purchased from Echelon Bioscience (Salt Lake City, UT, USA). All other reagents were purchased from Sigma-Aldrich (St. Louis, MO).

### Animals

Male B6.129S4-Itgam^tm1Myd^/J (MAC1^**-/-**^, on a C57BL/6J background) and C57BL/6J (MAC1^+/+^) mice were purchased from Jackson Laboratories (Bar Harbor, Maine). All the animals were housed in a specific pathogen free facility, and fed a standard laboratory chow *ad libitum*. Housing, breeding and experimental use of the animals were performed in strict accordance with the National Institutes of Heath guidelines.

### Aβ preparation

The peptide (1-42) was reconstituted in PBS, pH 7.4 at a concentration of 100 μM. Aliquots of stocks were incubated at 37°C for 3 days to form amyloid fibrils.

### Aβ injection in vivo

Male MAC1^-/- ^and MAC1^+/+ ^mice were anesthetized with sodium pentobarbital (80 mg/kg) and positioned in a small animal stereotaxic apparatus. Injection of Aβ into SN was made using the flowing stereotaxic coordinates, measured from bregma: 3.0 mm posterior, 1.7 mm lateral, and 4.7 mm ventral. Aβ (2 μg in 1 μl of saline) was injected into the right side of the SN over a period of 2 min, and the injection needle was kept in place for 2 min after the injection. A control injection of saline alone was made into the left side of SN under the conditions described above. Ten animals were used for each strain.

### Analysis of neurotoxicity

For the *in vivo *study, 24 consecutive coronal brain slices (35-μm thickness), which encompassed the entire SN, compact part, were collected. A normal distribution of the number of TH-positive neurons in the SN, compact part was constructed based on the counts of 24 slices from MAC1^-/- ^mice and MAC1^+/+ ^mice. The distribution curves from these two noninjected groups superimpose and show no difference in number and shape of the curves. Eight evenly spaced brain slices from saline or Aβ-injected animals were immunostained with an antibody against TH and subsequently TH-positive neurons were counted. The distribution of the cell numbers from each animal was matched with the normal distribution curve to correct for errors resulting from the cutting of the brain. Three individuals performed counting in a double-blind manner. Conclusions were drawn only when the difference was within 5%.

### Primary mesencephalic mixed neuron-glia cultures

Primary mesencephalic mixed neuron-glia cultures were prepared from the brains of embryonic day 13 ± 0.5 MAC1^+/+ ^and MAC1^-/- ^mice as previously described [20]. Briefly, the ventral midbrain portion of the embryonic brain was dissected under a microscope and kept in cold minimum essential medium (MEM). Mesencephalic tissues were isolated and dissociated with gentle mechanical trituration. Cells were diluted to 1.5 × 10^6^/ml in maintenance medium (MEM supplemented with 10% heat-inactivated fetal bovine serum (FBS), 10% heat-inactivated horse serum, 1 g/L glucose, 2 mM L-glutamine, 1 mM sodium pyruvate, 100 μM non-essential amino acids, 50 U/ml penicillin, and 50 μg/ml streptomycinand) and seeded in 24-well culture plates pre-coated with poly-D-lysine (20 μg/ml). Plates were maintained at 37°C in a humidified atmosphere of 5% CO_2 _and 95% air. Seven day-old cultures were used for treatment. The composition of the cells at the time of treatment was approximately 48% astrocytes, 11% microglia, 40% neurons with 1% of which being TH-positive neurons.

### Primary mesencephalic microglia-depleted cultures

Twenty-four hours after seeding the cells, 1 mM L-leucine methyl ester was added to the culture. Two days later, cultures were changed back to maintenance medium and were used for treatment 7 days after initial seeding. The cultures stained with Iba-1 antibody showed less than 0.1% microglia.

### Primary cortical neuron-glia culture

Mouse cortical neuron-glia were prepared from the brains of embryonic day 16 ± 0.5 MAC1^+/+ ^and MAC1^-/- ^mice. The cortex was dissociated by mild mechanical trituration in MEM. After pelleting by centrifugation, cells were resuspended and plated (5 × 10^5^/well) in 24-well culture plates precoated with poly d-lysine. Cultures were maintained in the same medium as described above for mesencephalic neuron-glia cultures. Seven-day cultures were used for treatment. The composition of cortical neuron-glia cultures was determined by immunostaining with antibodies against Neu-N and Iba-1. Cortical neuron-glia cultures contained 60% Neu-N-IR neurons and 3.1% Iba-1-IR microglia. The remaining cells were presumed to be astroglia.

### Primary microglia-enriched cultures

Primary microglia-enriched cultures were prepared from the whole brains of 1-day-old MAC1^+/+ ^and MAC1^-/- ^pups as described previously [20]. Briefly, brain tissues were triturated after removing the meninges and blood vessels. Cells were seeded at 5 × 10^7 ^in a 150 cm^3 ^cultures flask with Dulbecco's modified Eagle's medium/F12 (DMEM/F12) containing 10% heat-inactivated FBS, 2 mM L-glutamine, 1 mM sodium pyruvate, 100 μM non-essential amino acids, 50 U/ml penicillin and 50 μg/ml streptomycin, pH = 7.2. After a confluent monolayer of glia cells had been obtained (12-14 days after initial seeding), microglia were shaken off, collected and seeded. The purity of microglia is greater than 98%.

### Uptake assays for [^3^H]dopamine or [^3^H]GABA

Cells were incubated for 15 min at 37°C with 1 μm[^3^H]DA or 5 μm[^3^H]GABA in Krebs-Ringer buffer (16 mm NaH_2_PO_4_, 16 mm Na_2_HPO_4_, 119 mm NaCl, 4.7 mm KCl, 1.2 mm MgSO_4_, 1.3 mm EDTA, pH 7.4). Non-specific uptake was blocked by 10 μm mazindol for dopamine uptake, or 10 μm NO-711 and 1 mmβ-alanine for GABA uptake. After washing the cells three times with ice-cold Krebs-Ringer buffer (1 ml/well) and lysing with 1 N NaOH (0.5 ml/well), the lysate was mixed with 15 mL scintillation fluid and radioactivity was determined with a liquid scintillation counter. The specific [^3^H]dopamine or [^3^H]GABA uptake was calculated by subtracting the amount of radioactivity obtained in the presence of mazindol or NO-711 and β-alanine from that obtained in the absence of mazindol or NO-711 and β-alanine.

### Immunostaining

Immunostaining was performed as described previously [20]. Dopaminergic neurons were stained with the antibody against TH (1:5000). Microglia were stained with the antibody raised against F4/80 (1:20) or Iba-1 (1:1000). In brief, brain sections or 3.7% formaldehyde fixed cultures were treated with 1% hydrogen peroxide followed by sequential incubation with blocking solution (PBS containing 1% BSA, 0.4% Triton-X 100 and 4% appropriate serum), primary antibody, biotinylated secondary antibody, and Vectastain ABC reagents. After washing, the bound complex was visualized by incubating cells with 3, 3`-diaminobenzidine and urea-hydrogen peroxide tablets dissolved in water. Color development was terminated by removal of the reagents and washing the cultures with PBS. Images of *in vitro *cultures or brain staining were recorded with an inverted microscope (Nikon, Tokyo, Japan) or Zeiss microscope (Zeiss, Germany), respectively, connected to a charge-coupled device camera (DAGE-MTI, Michigan City, IN, USA) operated with the MetaMorph software (Universal Imaging Corporation, Downingtown, PA, USA).

### Superoxide assay

The extracellular superoxide production was determined by measuring the superoxide dismutase (SOD)-inhibitable reduction of tetrazolium salt, WST-1 as described before [20] with modifications. Primary microglial enriched cultures grown in 96-well plates were treated with Aβ or vehicle in 150 μl of phenol red-free treatment medium. At 10 min after treatment, 50 μl of WST-1 (1 mM) in phenol red-free treatment medium, with or without SOD (600 units/ml), was added. The absorbance at 450 nm was read immediately with a Spectra Max Plus microtiter plate spectrophotometer (Molecular Devices, Sunnyvale, CA). For the superoxide assay using microglia-enriched culture, cells were plated at 1 × 10^5^/well in 96-well plate and incubated at 37°C in a humidified atmosphere of 5% CO_2 _and 95% air for 12 hours. The cells were washed twice and left in 100 μl HBSS. Fifty μl HBSS, PMA or Aβ were added. Then 50 μl of WST-1 (1 mM) in HBSS, with or without SOD (600 units/ml), was added. The absorbance at 450 nm was read immediately.

### Confocal microscopy

Enriched microglial cells from mice were seeded in dishes at 5 × 10^4 ^cells/well and treated with Aβ for 10 min. Cells were fixed with 3.7% paraformaldehyde in PBS for 10 min. After washing with PBS, cells were incubated with rabbit polyclonal antibody against PIP_3 _(0.5 μg/ml). Cells were then washed and incubated with FITC-conjugated goat anti-rabbit antibody. Focal planes spaced at 0.4 μm intervals were imaged with a Zeiss 510 laser scanning confocal microscope (63X PlanApo 1.4 numerical aperture objective) equipped with LSM510 digital imaging software. Intracellular reactive oxygen species (iROS) were assessed as follows. Enriched microglial cells from mice were seeded in dishes at 5 × 10^4 ^cells/well and were incubated with 1 × HBSS containing 5 μM dichlorodihydrofluorescein diacetate (DCFDA) in the dark for 1 hour. Then cells were treated with Aβ for 10 min. Fluorescent images were captured using Zeiss 510 laser scanning confocal microscope.

### Cell extract

Whole cell lysates from neuron-glia cultures were prepared with lysis buffer (Cell Signaling, Danvers, MA). Subcellular fractionation was performed. For subcellular fractions, microglia cells were lysed in hypotonic lysis buffer (1 mM EGTA, 1 mM EDTA, 10 mM β-glycerophosphate, 10 mM NaF, 1 mM sodium orthovanadate, 2 mM MgCl_2_, 10 mM dithiothreitol, 1 mM phenylmethylsulfonyl fluoride, and 10 μg/ml each of leupeptin, aprotinin, and pepstatin A), incubated on ice for 30 min, and then subjected to Dounce homogenization (20-25 stokes, tight pestle A). The lysates were loaded onto a sucrose gradient in lysis buffer (final 0.5 M) and centrifuged at 1,600 × g for 15 min. The supernatant above the sucrose gradient was used as the cytosolic fraction after centrifugation at 150,000 × *g *for 30 min. The pellets were solubilized in 1% Nonidet P-40 hypotonic lysis buffer and were used as membranous fraction.

### Western blot analysis

Equal amounts of protein (40 μg/lane) were separated by 4-12% Bis-Tris-polyacrylamide electrophoresis gel and transferred to polyvinylidene difluoride membranes (Novex, San Diego, CA). Membranes were blocked with 5% nonfat milk and incubated with primary antibody (rabbit anti-p47^*phox *^antibody (1: 2000), rabbit anti-GAPDH (1:2000), mouse anti-gp91^*phox *^(1:2000) or rabbit anti-Iba-1 (1:3000)) overnight at 4°C. Membrane was then incubated with horseradish peroxidase-linked anti-rabbit or mouse IgG (1:3000) for 1 h at 25°C. ECL Plus reagents (GE Healthcare, Little Chalfont, Buckinghamshire, UK) were used for detection.

### Statistical analysis

The data were expressed as mean ± SEM. Statistical significance between two groups was assessed with an analysis of variance followed by Student's *t*-test or nonparametric statistics. Statistical significance between multiple groups was performed using a one- or two-way analysis of variance (ANOVA). When ANOVA showed a significant difference, an LSD multiple comparisons post-hoc test was performed. A value of p < 0.05 was considered statistically significant.

## Results

### Aβ-induced dopaminergic and GABAergic neurotoxicity in neuron-glia cultures was attenuated with the absence of MAC1

**Figure 1:**
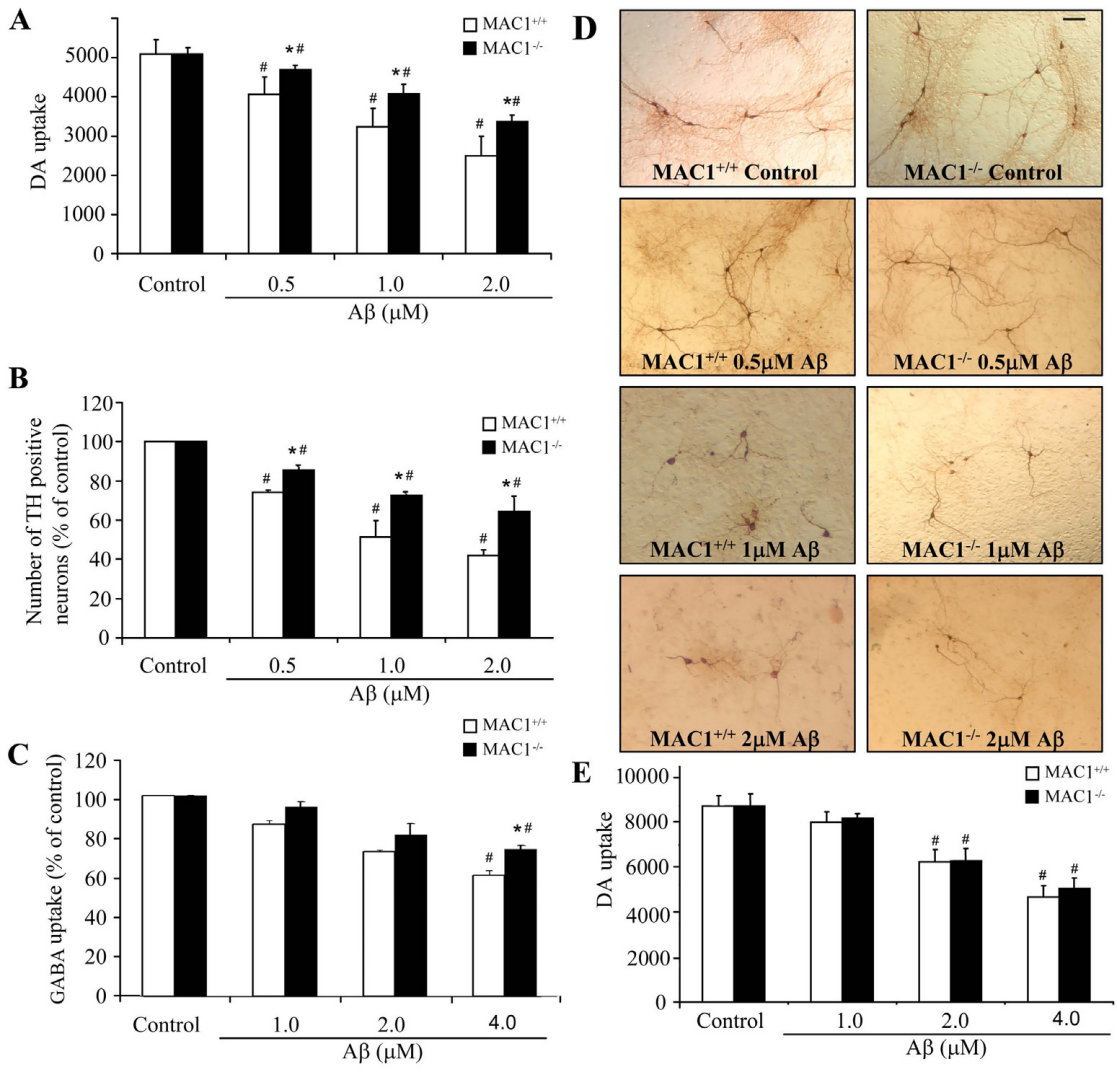
**Absence of MAC1 attenuates Aβ-induced dopaminergic and GABAergic neurotoxicity in neuron-glia cultures**. Mice (MAC1^+/+ ^and MAC1^-/-^) mesencephalic neuron-glia cultures in 24-well plates were treated with vehicle medium (control group) or 0.5 μM, 1.0 μM and 2.0 μM Aβ for 7 days. (A) Aβ-induced dopaminergic neurotoxicity was quantified by [^3^H] dopamine uptake assay. (B) Numbers of TH-positive cells remaining in the neuron-glia cultures after vehicle or Aβ treatment. (D) Representative light microscopic images are shown for TH-positive neurons treated with vehicle or Aβ. Scale bar: 100 μm. (C) Mice (MAC1^+/+ ^and MAC1^-/-^) cortical neuron-glia cultures in 24-well plates were treated with vehicle or 1.0 μM, 2.0 μM and 4.0 μM Aβ for 7 days. Aβ-induced GABAergic neurotoxicity was quantified by [^3^H] GABA uptake assay. (E) Mice (MAC1^+/+ ^and MAC1^-/-^) mesencephalic microglia-depleted cultures in 24-well plates were treated with vehicle medium (control group) or 1.0 μM, 2.0 μM and 4.0 μM Aβ for 7 days. Aβ-induced dopaminergic neurotoxicity was quantified by [^3^H] dopamine uptake assay. Results are from four independent experiments. #: p < 0.05 compared with corresponding vehicle-treated controls. *: p < 0.05 compared with MAC1^+/+ ^cultures after same treatments.

To investigate the role of MAC1 in Aβ-induced neurotoxicity, mesencephalic neuron-glia cultures prepared from MAC1^+/+ ^and MAC1^-/- ^mice were treated with vehicle (controls) or 0.5, 1 or 2 μM Aβ. Dopaminergic neurotoxicity was assessed by dopamine uptake assay and cell number count/morphological analysis of immunostained TH-positive neurons. Aβ exposure caused concentration-dependent decreases in [^3^H] dopamine uptake (Figure 1A) and the number of TH-positive neurons (Figure 1B) in cultures from MAC1^+/+ ^and MAC1^-/- ^mice. For both measurements, decreases were significantly less in the MAC1^-/- ^cultures. Morphological analysis showed that TH-positive neurons in the Aβ-treated MAC1^+/+ ^cultures (Figure 1C) displayed much shorter and less elaborate neurites compared with those from MAC1^-/- ^cultures. We also tested Aβ-induced GABAergic neurotoxicity in cortical neuron-glia cultures. We found that Aβ exerted a less potent reduction in GABA uptake in cortical neuron-glia cultures than DA uptake in mesencephalic neuron-glia cultures (Figure 1D). Because there are less microglia presented in cortical neuron-glia cultures than in mesencephalic neuron-glia cultures (about 3% to 10%), these results indicate that the presence of glia might potentiate the toxic effect of Aβ and that is the reason that we used mesencephalic cultures for the rest of the study. Besides, we found no difference in Aβ-induced neurotoxicity in microglia-depleted cultures prepared from MAC1^+/+ ^and MAC1^-/- ^mice (Figure 1E), confirming the role of microglial MAC1 in the different dopaminergic neurotoxicity of Aβ that we observed in neuron-glia cultures.

### MAC1 is necessary for Aβ-induced microglial activation

**Figure 2:**
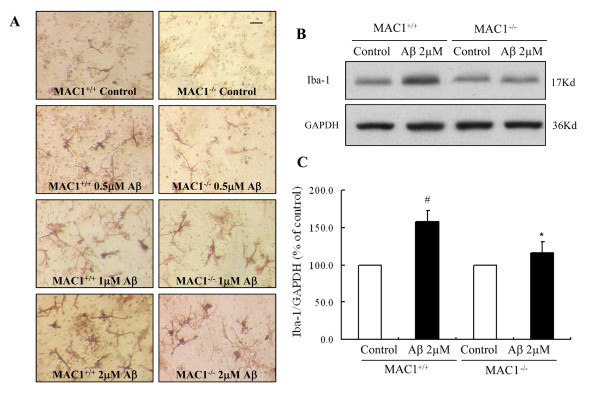
**MAC1 is necessary for Aβ-induced microglial activation**. Mesencephalic neuron-glia cultures from MAC1^+/+ ^and MAC1^-/- ^mice were treated with vehicle medium (control group) or Aβ for 2 days. (A) Cultures were fixed after treatments. Microglia were visualized by immunostaining of the F4/80 antigen, a microglial marker. The images presented are representative of three independent experiments. Scale bar: 50 μm. (B) Western blot analysis of microglial activation. Cell lysates of cultures from MAC1^+/+ ^and MAC1^-/- ^mice were prepared 2 days after vehicle or 2.0 μM Aβ treatment and immunoblot analysis was performed for the measurement of Iba1 antigen. GAPDH was used as loading control. (C) The ratio of densitometry values of Iba1 and GAPDH was analyzed and normalized to respective control. Results are presented as mean ± SEM for three independent experiments. #: p < 0.05 compared with corresponding vehicle-treated controls. *: p < 0.05 compared with MAC1^+/+ ^cultures after same treatments.

After demonstrating the important role of MAC1 in Aβ-induced neurotoxicity, we continued to investigate whether MAC1 expression affects Aβ-induced microglial activation. The total microglial number in the cultures from MAC1^+/+ ^and MAC1^-/- ^mice are comparable (data not shown). Activation of microglia was assessed using F4/80 immunostaining in MAC1^+/+ ^and MAC1^-/- ^neuron-glia cultures following exposure to Aβ. One day after treatment with Aβ, numerous activated microglia, characterized by intensified F4/80 staining and enlarged cell size, were distributed throughout the MAC1^+/+ ^culture (Figure 2A); MAC1^+/+ ^cultures contained a mixture of process-bearing ramified microglia and process-free amoeboid-like microglia. With 2 μM Aβ, MAC1^+/+ ^cultures exhibited more F4/80-positive cells that appeared to be fully activated microglia with an amoeboid and condensed morphology. In cultures from MAC1^-/- ^mice, Aβ-induced activation of microglia was noticeably less pronounced (Figure 2A). Western blots using antibody for Iba-1, a widely used marker for microglial activation, showed elevated Iba-1 expression in Aβ-treated MAC1^+/+ ^cultures but not in MAC1^-/- ^cultures (Figure 2B and 2C).

### *MAC1*^***-/- ***^*mice exhibit reduced Aβ-induced nigral dopamine neurotoxicity and microglial activation in vivo*

**Figure 3:**
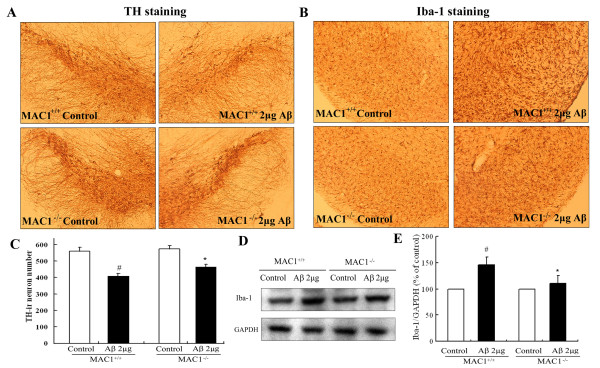
**Dopaminergic neurons from MAC1-deficient mice are more resistant to Aβ-induced neurotoxicity *in vivo***. Two μg of Aβ were injected into the right side of adult MAC1^+/+ ^and MAC1^-/- ^mouse SN; vehicle saline alone (control group) was injected into the left side. After 7 days, brains were removed, dopaminergic neurons were stained with an antibody against TH and the microglia were stained with an antibody against Iba-1. Ten animals were used for each group. Dopaminergic neurons were counted in a double-blind manner by three individuals. (A**) **Immunocytochemical analysis of TH-positive neurons. (B**) **Number of TH-positive neurons. (C**) **Immunocytochemical analysis of microglia. (D) Western blot analysis of microglial activation in midbrain area. GAPDH was used as loading control. (E) The ratio of densitometry values of Iba1 and GAPDH was analyzed and normalized to respective control. Results are presented as mean ± SEM. #: p < 0.05 compared with corresponding saline-treated side. *: p < 0.05 compared with MAC1^+/+ ^mice after same treatments.

To determine the role of MAC1 in Aβ-induced DA neurotoxicity *in vivo*, we stereotaxically injected Aβ (2 μg in 1 μl of saline) into the substantia nigra (SN) of MAC1^+/+ ^and MAC1^-/- ^mice. Seven days later, the brains were removed, and coronal sections through the nigral complex were immunostained with an antibody against tyrosine hydroxylase (TH), a marker for dopaminergic neurons. Although TH-positive neuron counts were comparable in the saline-injected sides of MAC1^+/+ ^mice and MAC1^-/- ^mice, Aβ injection caused a greater loss of TH-positive neurons in the SN of MAC1^+/+ ^mice than in MAC1^-/- ^mice (Figure 3A). Cell counts revealed a 27% loss of DA neurons in MAC1^+/+ ^controls and 17% loss in MAC1^-/- ^mutants (Figure 3C). Thus, deletion of MAC1 protected neurons against microglial activation *in vivo*.

Microglial morphological changes after Aβ injection were also examined in the SN (Figure 3B). Microglia were stained with an antibody against Iba1. Resident microglia in both MAC1^+/+ ^and MAC1^-/- ^mice express a basal level of Iba1, appear small and bear thin processes. Microglial activation, as demonstrated by a dramatic increase in Iba1 staining, was highly visible in the SN of MAC1^+/+ ^mice post-Aβ injection. Numerous microglia assumed a highly activated amoeboid state with an enlarged cell body and thicker cellular processes compared with corresponding saline-injected control sides. Aβ-induced activation of microglia was less pronounced in the SN of MAC1^-/- ^mice, which exhibited minimal morphological changes. We also obtained the quantitative changes of Iba-1 in midbrain area by western blot assay. The results showed elevated Iba-1 expression in Aβ-treated MAC1^+/+ ^mice but not in MAC1^-/- ^mice (Figure 3D and 3E). These *in vivo *findings confirmed our *in vitro *result indicating that MAC1 plays a role in Aβ-induced microglial activation and subsequent loss of dopaminergic neurons in the SN.

### Aβ-induced ROS production from microglia is reduced in MAC1^-/- ^cultures

**Figure 4:**
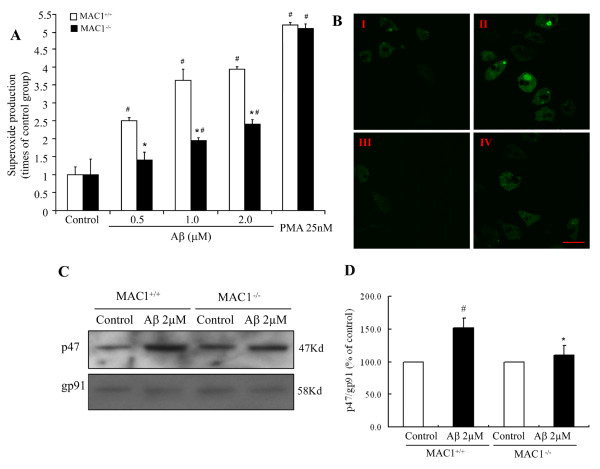
**MAC1 mediates activation of PHOX and production of superoxide**. (A) Microglia-enriched cultures from MAC1^+/+ ^and MAC1^-/- ^mice were treated with vehicle medium (control group), Aβ or PMA for 10 min. Extracellular superoxide generation was measured by the SOD-inhibitable reduction of tetrazolium salt, WST-1. (B) Microglia-enriched cultures from MAC1^+/+ ^and MAC1^-/- ^mice were incubated with HBSS containing 5 μM DCFDA in the dark for 1 hour. Then cells were treated with vehicle medium (control group) or Aβ for 10 min. Fluorescent images were captured using Zeiss 510 laser scanning confocal microscope. Scale bar: 25 μm. (C) Western blot assays for p47^*phox *^levels in membrane fractions of microglia from MAC1^+/+ ^and MAC1^-/- ^mice 10 min after vehicle medium (control group) or Aβ treatment. (D) Densitometry analysis was performed with values of p47^*phox *^normalized to loading control and further normalized to control levels. Data are presented as mean ± SEM from three independent experiments. #: p < 0.05 relative to corresponding vehicle-treated control cultures. *: p < 0.05 relative to MAC1^+/+ ^cultures after same treatments.

We previously showed that microglia enhance Aβ-induced toxicity by producing reactive oxygen species, especially extracellular superoxide [6]. To determine the role of MAC1 in Aβ-induced superoxide production, enriched microglial cultures from MAC1^+/+ ^and MAC1^-/- ^mice were exposed to 0.5, 1 and 2 μM Aβ and superoxide production was measured. Figure 4A shows that Aβ increased extracellular superoxide in both cultures. However, the increase was significantly reduced (P < 0.05) in MAC^-/- ^cultures, indicating a role for MAC1 in Aβ-induced superoxide production. To ensure the normal function of PHOX itself in MAC1^-/- ^mice, microglia-enriched cultures from MAC1^+/+ ^and MAC1^-/- ^mice were treated with vehicle, PMA, a potent protein kinase C agonist that can directly induce the production of superoxide catalyzed by PHOX. Although Aβ induced significantly less production of superoxide in MAC1^-/- ^microglia compared with that of MAC1^+/+ ^microglia, MAC1^+/+ ^and MAC1^-/- ^microglia demonstrated the same capacity to produce superoxide after PMA, indicating an intact capability for PHOX-mediated superoxide production in these two genotypes. This suggests that the reduced production of superoxide observed in MAC1^-/- ^neuron-glia cultures after Aβ exposure cannot be attributed to the malfunction of PHOX.

We also measured intracellular ROS production induced by Aβ using DCFDA. Enriched microglial cells from mice were seeded in dishes at 5 × 10^4 ^cells/well and were incubated with 1 × HBSS containing 5 μM DCFDA in the dark for 1 hour. Then cells were treated with Aβ for 10 min. This non-fluorescent compound is oxidized by intracellular ROS to 2,7-dichlorodihydrofluorescein (DCF), which is fluorescent. DCF fluorescence stimulated by Aβ was detected using laser confocal scanning microscopy (Figure 4B). No DCF fluorescence was detected in control cells from both MAC1^+/+ ^and MAC1^-/- ^cultures (Figure 4B I is MAC1^+/+ ^control culture and Figure 4B III is MAC1^-/- ^control culture). Aβ exposure increased DCF fluorescence intensity in both cultures, but the increase in MAC1^-/- ^cultures was much lower (Figure 4B II and Figure 4B IV are MAC1^+/+ ^culture and MAC1^-/- ^culture treated with Aβ, respectively.). Thus, MAC1 is closely linked with Aβ-induced extracellular and intracellular ROS production in microglia.

### MAC1 is critical for Aβ-induced activation of PHOX and subsequent ROS production after Aβ treatment

Since extracellular superoxide is an important factor mediating MAC1-enhanced Aβ neurotoxicity [6] and since PHOX is the key enzyme in extracellular superoxide generation in microglia, we investigated whether PHOX activation depended upon MAC1 expression. Activation of PHOX requires the translocation of cytoplasmic subunits (p47^*phox*^, p67^*phox*^, p40^*phox*^, and Rac) and their subsequent interaction with the membrane-spanning flavocytochrome b558 [21]. To determine whether MAC1 played a role in PHOX activation, membrane translocation of p47^*phox *^was monitored in microglial cultures by western blot following Aβ exposure. We found significantly increased p47^*phox *^levels in the membrane fraction of MAC1^+/+ ^microglia, but not MAC1^-/- ^microglia 10 minutes after Aβ exposure (Figure 4C&4D). In contrast, after Aβ exposure, membrane content of p47^*phox *^in the MAC1^-/- ^microglia was substantially reduced. These results suggest the involvement of MAC1 in p47^*phox *^membrane translocation and thus PHOX activation induced by Aβ.

### Aβ activates PHOX via PI3K signaling

**Figure 5:**
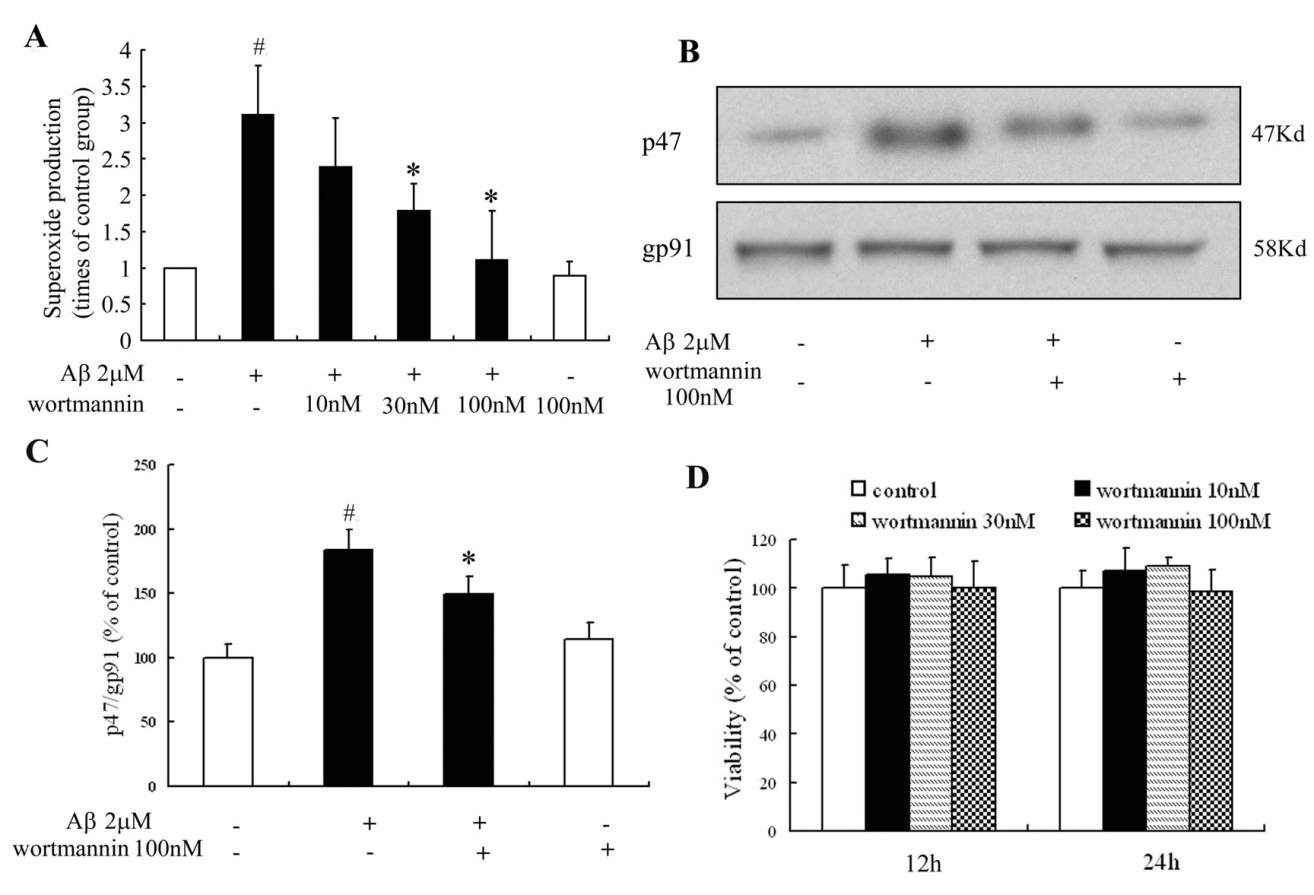
**PI3K mediates signaling between MAC1 receptor and PHOX**. Microglia-enriched cultures from MAC1^+/+ ^mice were pretreated with wortmannin for 30 min and then challenged with Aβ for 10 min. (A) Extracellular superoxide generation was measured by the SOD-inhibitable reduction of tetrazolium salt, WST-1. (B) Western blot assays for p47^*phox *^levels in membrane fractions from microglia. (C) Densitometry analysis was performed with values of p47^*phox *^normalized to loading control and further normalized to control levels. (D) Microglia-enriched cultures from MAC1^+/+ ^mice were treated with vehicle medium (control group) or different concentrations of wortmannin for 12 or 24 hours; cell viability was measured using MTT assay. Data are presented as mean ± SEM for three independent experiments. #: p < 0.05 relative to vehicle-treated control cultures. *: p < 0.05 relative to Aβ-treated cultures.

A recent study has shown that specific interactions between phox homology (PX) domains and phosphoinositides play a crucial role in the regulation of PX-domain-containing proteins, and has identified the PX domain as a phosphoinositide-binding module involved in cellular signal transduction [22]. We hypothesized that PI3K mediates Aβ-induced PHOX activation through the PX domains in two subunits of the NADPH oxidase, p47^*phox *^and p40^*phox *^[23]. Microglial enriched cultures from MAC1^+/+ ^mice were pre-treated with wortmannin, a potent PI3K inhibitor for 30 min after which the cultures were challenged with Aβ. Figure 5A shows that wortmannin reduced Aβ-induced superoxide production from MAC1^+/+ ^microglia in a concentration-dependent manner; wortmannin alone did not alter superoxide production nor did it induce microglial toxicity (Figure 5D). Western blot analysis showed 100 nM wortmannin significantly inhibited the Aβ-induced increase in p47^*phox *^levels in the membrane fraction of MAC1^+/+ ^cultures (Figure 5B, C), indicating suppression of PHOX activation. These results indicate that PI3K activation is an upstream signal in Aβ-induced PHOX activation in microglia.

### MAC1 is necessary for PI3K activation by Aβ

**Figure 6:**
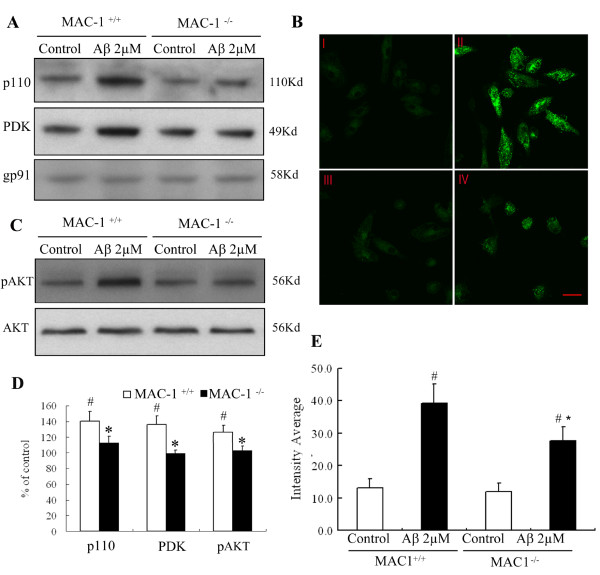
**MAC1 is necessary for PI3K activation elicited by Aβ**. (A) Western blot assays for p110 and PDK levels in membrane fractions of microglia from MAC1^+/+ ^and MAC1^-/- ^mice 10 min after vehicle medium (control group) or Aβ treatment. (B) Enriched microglial cells from MAC1^+/+ ^and MAC1^-/- ^mice were treated with vehicle medium (control group) or Aβ for 10 min, fixed and permeabilized. I and II are MAC1^+/+ ^cultures treated with vehicle or Aβ, respectively. III and IV are MAC1^-/- ^cultures treated with vehicle or Aβ, respectively. (C) Western blot assays for pAKT and AKT levels in microglia from MAC1^+/+ ^and MAC1^-/- ^mice 10 min after vehicle medium (control group) or Aβ treatment. (D) Densitometry was performed with values normalized to respectively loading control and further normalized to control levels. Cells were incubated with a rabbit polyclonal antibody against PIP_3 _and then with a FITC-conjugated goat anti-rabbit antibody. Shown are representative confocal images. Scale bar: 25 μm. (E) Quantitative analysis of the figures in 6B. Average cellular fluorescence was quantitated from at least 100 separate cellular measurements obtained from each treatment group. Background fluorescence, determined in the absence of fluorescence labeled antibody, was minimal in both cell populations (data not shown). Experiments were performed at least three times. #: p < 0.05 relative to corresponding vehicle-treated control cultures. *: p < 0.05 relative to MAC1^+/+ ^cultures after same treatments.

Having demonstrated involvement of PI3K in Aβ-induced activation of PHOX, we examined the role of MAC1 in Aβ-induced PI3K activation. Translocation of p110 catalytic subunit of type I PI3K to the membrane is required for PI3K activation. Western blots showed that a 10 min Aβ exposure of MAC1^+/+ ^cultures increased the membrane localization of p110 (Figure 6A). In contrast, Aβ-induced p110 translocation to the membrane was significantly suppressed in MAC1^-/- ^cultures. Figure 6B shows the confocal microscopy results of PIP_3 _staining before and after Aβ challenge using a monoclonal antibody specific for PIP_3_, which is a product of activated PI3K. In MAC1^+/+ ^microglial cultures, Aβ increased PIP_3 _staining within 15 min and the positive staining was localized mainly in the cytoplasm and perinuclear, indicating the activation of PI3K. Similar to the p110 translocation result, Aβ-induced increase in PIP_3 _staining in MAC1^-/- ^microglial cultures was much reduced (Figure 6E).

To confirm a role for MAC1 in Aβ-induced PI3K activation, we measured activation of PDK1 and AKT, two kinases downstream of PI3K. Activated PI3K produces second messenger PIP_3_, which binds to the pleckstrin homology domain of PDK1, recruiting it to the plasma membrane where PDK1 is activated by phosphorylation of its activation loop residue Ser^241 ^[22]. Aβ exposure of of MAC1^+/+ ^microglial cultures induced the phosphorylation of the membrane-associated PDK1 and AKT within 15 min. Significantly less p-PDK and pAKT were found in MAC1^-/- ^microglial cultures (Figure 6C and 6D). Taken together, these data indicate that MAC1 is upstream of PI3K in Aβ-induced activation of microglia.

## Discussion

**Figure 7:**
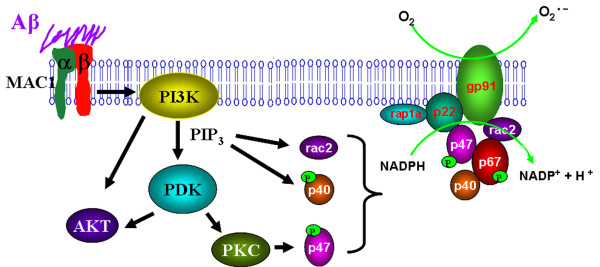
**β-amyloid signals through microglial MAC1 receptor and PI3K to PHOX**. Microglia exposed to Aβ exhibit a respiratory burst leading to the release of superoxide anion (O_2_^-^). Release of superoxide is mediated in part through the Aβ cell surface receptor MAC1. Aβ engagement of MAC1 receptor leads to the initiation of complex signaling events mediated by PI3K activation and PIP_3 _production, which leads to AKT phosphorylation and activation of PDK. Activation of these signaling cascades is linked to the activation of PHOX. PHOX plays an essential role in innate immunity by catalyzing the formation of superoxide. PHOX consists of two integral membrane proteins, p22^*phox *^and gp91^*phox*^, which together form a heterodimeric flavoprotein known as cytochrome b558. In addition, there are four cytosolic components p47^*phox*^, p67^*phox*^, p40^*phox*^, and the small G-protein Rac. As an important component for PHOX activation, the GDP/GTP exchange on Rac-1 is reported to be a point of possible PI3K intervention. PIP_3 _is reported to bind to p47^*phox *^and p40^*phox*^, and mediates their phosphorylation[46]; PI3K signaling pathway may also sometimes be involved in PKC activation, thus play an important role in the phosphorylation of p47^*phox*^. The cytosolic components then translocate to the membrane where they form a complex with cytochrome b558. The oxidase complex then initiates electron flow and generation of superoxide through the NADPH-derived electron reduction by the flavocytochrome. These findings suggest that MAC1 and PI3K are involved in upstream signaling cascades responsible for activating PHOX assembly and microglia in response to Aβ.

We demonstrate here that Aβ potently activates microglia in part through the activation of MAC1 receptors, followed by the production of superoxide free radicals, and leading to neuronal death. These results not only confirm our earlier observations that the presence of microglia potentiated the neurotoxicity of Aβ, but also reveal several novel findings that illustrated in Figure 7: (1) a role for MAC1 receptor in Aβ-induced microglial activation, (2) involvement of MAC1 receptor in PHOX activation which mediates Aβ-induced neurotoxicity and (3) involvement of PI3K in PHOX activation signals by MAC1 receptor.

In the mature brain, microglia are readily activated in response to certain cues such as brain injury or immunological stimuli. Activated microglia serve diverse beneficial functions essential to neuronal survival [24,25]. However, with chronic and over-activation, microglia themselves can be detrimentally neurotoxic by excessive production of a large array of cytotoxic factors such as superoxide [26,27]. The signals responsible for microglial dysregulation are diverse, ranging from exposure to environmental toxins to neuronal death due to brain damage or genetic diseases.

There has been considerable interest from several laboratories in identifying microglial surface receptors that interact with Aβ fibrils. Scavenger receptor class A (SR-A), scavenger receptor-BI (SR-BI) and CD36 [28-30]El Khoury et al., 1996 J. El Khoury, S.E. Hickman, C.A. Thomas, L. Cao, S.C. Silverstein and J.D. Loike, Scavenger receptor-mediated adhesion of microglia to beta-amyloid fibrils, Nat andand and other receptors such as macrophage colony-stimulating factor receptor (MCSF) [31], formyl peptide receptor-1 receptor (FPR-1) [32], receptor for advanced glycation end products (RAGE) [33], etc have been reported to be the binding sites for Aβ. A series of papers from Dr. Landreth et al. show that Aβ interacts with a microglial surface receptor complex composed of B-class scavenger receptor CD36, integrin-associated protein/CD47, and α_6_β_1_-integrin [34-37].

MAC1 is a leukocyte integrin expressed on microglia and it is a versatile adhesion and recognition receptor. MAC1 plays a critical role in microglial migration via pathways that include kinase cascades and cytoskeleton rearrangements, culminating in activation critical for antimicrobial leukocyte functions. MAC1 is also reported to cooperatively activate other key adhesion and defense receptors [38,39]. In addition to above-mentioned functions, recent work from our laboratory has demonstrated a critical role of MAC1 in mediating the reactivation of microglia in response to neuronal damage/death, so-called reactive microgliosis [17-19]. Our studies suggest that MAC1 serves as a pattern recognition receptor mediating the neurodegeneration produced by toxic substances released from damaged or dead neurons. Since Aβ is one of the constituents in Lewy body, damaged dopaminergic neurons may release Aβ to the extracellular space and further activate microglia at least partially through pattern recognition receptor. Results from this study lend additional support to this possibility.

In the current study, we revealed that MAC1 deficiency mitigated the loss of dopaminergic neurons and GABAergic neurons induced by Aβ exposure, which indicates that MAC1 is critical in the Aβ-induced neurotoxic process. MAC1 deficiency did not change the sensitivity of neurons per se because Aβ exerted same degree of toxicity in neuron-enriched cultures from MAC1^-/- ^and MAC1^+/+ ^mice. The presence of microglia with functional MAC1 expression was essential for the differential sensitivity to Aβ neurotoxicity. This conclusion is further supported by the lack of robust microglia activation in neuron-glia cultures from MAC1^-/- ^mice and the *in vivo *study using MAC1^-/- ^mice, as measured by changes in cell morphology and by the release of pro-inflammatory factors.

In microglia, activated PHOX is a major source of ROS. Most PHOX products are released into the extracellular space, and it is believed that extracellular ROS plays a major role in neurotoxicity associated with microglial activation [20]. Previous studies in our lab have shown the involvement of PHOX in microglial activation and associated neurotoxicity induced by Aβ [6]. The neurotoxicity observed in cultures from wild type mice that induced by Aβ was potently attenuated in cultures from PHOX^-/- ^mice. Our previous findings, together with present results showing that involvement of MAC1 in Aβ-induced neurotoxicity, lend credence to the conclusion that MAC1 receptors are coupled to PHOX for enzymatic activation and subsequent production of superoxide. These conclusions are further supported by the fact that Aβ-induced translocation of p47^*phox *^from cytosol to cell membrane is substantially diminished in MAC1^-/- ^cultures. This conclusion is consistent with earlier reports of MAC1-mediated PHOX activation in neutrophils [8] and eosinophils [14].

The next question addressed the signaling pathways mediating the coupling the activation between MAC1 and PHOX. As illustrated in Figure 7, our studies reveal that PI3K is a key mediator for MAC1 to signal the activation of PHOX. PI3K is a class of phosphate kinase that has been implicated in cell signaling pathways that affect cellular death and longevity as well as many other processes that have medically important implications related to disease states [40-42]. The preferred substrate of class I PI3K is phosphoinositide(4,5)bisphosphate (PIP_2_). Phosphorylation of PIP_2 _by PI3K generates PIP_3_, which is an important second messenger that can promote cell survival, growth, protein synthesis, mitosis, and motility [43,44]. Numerous studies showed that PI3K and PIP_3 _are involved in the regulation of PHOX. As an important component for PHOX activation, Rac-1 is reported to be a point of possible PI3K intervention [45]. PIP_3 _is believed to bind to p47^*phox *^and P40^*phox *^[46]. PI3K signaling pathway may also be involved in PKC activation, thus play an important role in the phosphorylation of p47^*phox *^[47,48]. The present results not only show the involvement of PI3K in Aβ-induced PHOX activation, but also suggest that PI3K lies downstream of MAC1 receptor (Figure7).

Although strong evidence from this study illustrates a critical role for MAC1 receptors in mediating Aβ-induced neurotoxicity, the inhibition of both dopaminergic neuronal loss and production of superoxide in MAC1-deficient mice or microglia was only partial. These findings indicate that other mechanisms are also involved in Aβ-induced effects. In fact, reports from Dr. Landreth's group recently showed that CD14 and Toll-like receptors 2 and 4 are required for fibrillar Aβ-stimulated microglial activation [49].

MAC1 has been reported to bind several cell surface and soluble ligands including iC3b, ICAM, fibrinogen, factor X, filamentous hemagglutinin, lipophosphoglycan, and LPS [50,51].12. M. Diamond, J. Garcia-Aguilar, J. Bickford, A. Corbi and T. Springer, The I domain is a major recognition site on the leukocyte integrin Mac-1 for four distinct adhesion ligands. J. Cell Biol. It has been demonstrated that MAC1 is colocalized with Aβ plaques in the brain, thus, it is highly likely that Aβ activated microglia partly through the binding to MAC1 receptors [52]. To confirm this hypothesis, our ongoing project is to determine if there is direct binding of Aβ to MAC1. Preliminary data from binding assays and immunoprecipitation assays both favor our hypothesis that Aβ does bind to MAC1 receptors.

In conclusion, results from this study indicate that MAC1 receptor is involved in Aβ-induced microglial activation and subsequent neurotoxicity. The present findings are important because they identify an early, possibly initiating, event at the microglial plasma membrane. There are substantial interests in identifying microglial surface molecules that serve in effect as pattern recognition receptors transducing the neurodegenerative effects of substances released from damaged or dead neurons. Interactions involving these putative microglial pattern recognition receptors may represent the initial step in reactive microgliosis, leading to progressive neuronal death in neurodegenerative diseases. In this regard, MAC1 may provide a target for therapeutic intervention. Development of specific MAC1 receptor antagonists may reduce the activation of the MAC1-PHOX complex and the subsequent production of proinflammatory factors, extending neuronal survival.

## Competing interests

The authors declare that they have no competing interests.

## Authors' contributions

DZ performed majority of the experiments and drafted the manuscript. XMH, LQ, SHC and HZ participated in the experiments. BW carried out the immunostaining assay. DSM and JSH conceived the study and its design and helped to draft the manuscript. All authors read and approved the final manuscript.

## References

[B1] Arai H Lee VM Hill WD Greenberg BD Trojanowski JQ Lewy bodies contain beta-amyloid precursor proteins of Alzheimer's disease Brain Res 1992 585 386 390 10.1016/0006-8993(92)91242-7 1511324

[B2] Yoshida T Ohno-Matsui K Ichinose S Sato T Iwata N Saido TC Hisatomi T Mochizuki M Morita I The potential role of amyloid beta in the pathogenesis of age-related macular degeneration J Clin Invest 2005 115 2793 2800 10.1172/JCI24635 16167083 PMC1201663

[B3] Jendroska K Kashiwagi M Sassoon J Daniel SE Amyloid beta-peptide and its relationship with dementia in Lewy body disease J Neural Transm Suppl 1997 51 137 144 9470134 10.1007/978-3-7091-6846-2_11

[B4] Haga S Akai K Ishii T Demonstration of microglial cells in and around senile (neuritic) plaques in the Alzheimer brain. An immunohistochemical study using a novel monoclonal antibody Acta Neuropathol 1989 77 569 575 10.1007/BF00687883 2750476

[B5] Itagaki S McGeer PL Akiyama H Zhu S Selkoe D Relationship of microglia and astrocytes to amyloid deposits of Alzheimer disease J Neuroimmunol 1989 24 173 182 10.1016/0165-5728(89)90115-X 2808689

[B6] Qin L Liu Y Cooper C Liu B Wilson B Hong JS Microglia enhance beta-amyloid peptide-induced toxicity in cortical and mesencephalic neurons by producing reactive oxygen species J Neurochem 2002 83 973 983 10.1046/j.1471-4159.2002.01210.x 12421370

[B7] Akiyama H McGeer PL Brain microglia constitutively express beta-2 integrins J Neuroimmunol 1990 30 81 93 10.1016/0165-5728(90)90055-R 1977769

[B8] Coxon A Rieu P Barkalow FJ Askari S Sharpe AH von Andrian UH Arnaout MA Mayadas TN A novel role for the beta 2 integrin CD11b/CD18 in neutrophil apoptosis: a homeostatic mechanism in inflammation Immunity 1996 5 653 666 10.1016/S1074-7613(00)80278-2 8986723

[B9] Perera PY Vogel SN Detore GR Haziot A Goyert SM CD14-dependent and CD14-independent signaling pathways in murine macrophages from normal and CD14 knockout mice stimulated with lipopolysaccharide or taxol J Immunol 1997 158 4422 4429 9127007

[B10] Akiyama H Tago H Itagaki S McGeer PL Occurrence of diffuse amyloid deposits in the presubicular parvopyramidal layer in Alzheimer's disease Acta Neuropathol 1990 79 537 544 10.1007/BF00296114 2183538

[B11] Liberatore GT Jackson-Lewis V Vukosavic S Mandir AS Vila M McAuliffe WG Dawson VL Dawson TM Przedborski S Inducible nitric oxide synthase stimulates dopaminergic neurodegeneration in the MPTP model of Parkinson disease Nat Med 1999 5 1403 1409 10.1038/70978 10581083

[B12] Le Cabec V Carreno S Moisand A Bordier C Maridonneau-Parini I Complement receptor 3 (CD11b/CD18) mediates type I and type II phagocytosis during nonopsonic and opsonic phagocytosis, respectively J Immunol 2002 169 2003 2009 12165526 10.4049/jimmunol.169.4.2003

[B13] Ross GD Vetvicka V CR3 (CD11b, CD18): a phagocyte and NK cell membrane receptor with multiple ligand specificities and functions Clin Exp Immunol 1993 92 181 184 10.1111/j.1365-2249.1993.tb03377.x 8485905 PMC1554824

[B14] Lynch OT Giembycz MA Barnes PJ Hellewell PG Lindsay MA 'Outside-in' signalling mechanisms underlying CD11b/CD18-mediated NADPH oxidase activation in human adherent blood eosinophils Br J Pharmacol 1999 128 1149 1158 10.1038/sj.bjp.0702892 10578126 PMC1571741

[B15] Ingalls RR Arnaout MA Delude RL Flaherty S Savedra R Jr Golenbock DT The CD11/CD18 integrins: characterization of three novel LPS signaling receptors Prog Clin Biol Res 1998 397 107 117 9575552

[B16] Medvedev AE Flo T Ingalls RR Golenbock DT Teti G Vogel SN Espevik T Involvement of CD14 and complement receptors CR3 and CR4 in nuclear factor-kappaB activation and TNF production induced by lipopolysaccharide and group B streptococcal cell walls J Immunol 1998 160 4535 4542 9574560

[B17] Hu X Zhang D Pang H Caudle WM Li Y Gao H Liu Y Qian L Wilson B Di Monte DA Macrophage antigen complex-1 mediates reactive microgliosis and progressive dopaminergic neurodegeneration in the MPTP model of Parkinson's disease J Immunol 2008 181 7194 7204 18981141 10.4049/jimmunol.181.10.7194 PMC2759089

[B18] Pei Z Pang H Qian L Yang S Wang T Zhang W Wu X Dallas S Wilson B Reece JM MAC1 mediates LPS-induced production of superoxide by microglia: the role of pattern recognition receptors in dopaminergic neurotoxicity Glia 2007 55 1362 1373 10.1002/glia.20545 17654704

[B19] Zhang W Dallas S Zhang D Guo JP Pang H Wilson B Miller DS Chen B Zhang W McGeer PL Microglial PHOX and Mac-1 are essential to the enhanced dopaminergic neurodegeneration elicited by A30P and A53T mutant alpha-synuclein Glia 2007 55 1178 1188 10.1002/glia.20532 17600340

[B20] Gao HM Liu B Zhang W Hong JS Critical role of microglial NADPH oxidase-derived free radicals in the in vitro MPTP model of Parkinson's disease Faseb J 2003 17 1954 1956 12897068 10.1096/fj.03-0109fje

[B21] Babior BM NADPH oxidase: an update Blood 1999 93 1464 1476 10029572

[B22] Lee JS Kim JH Jang IH Kim HS Han JM Kazlauskas A Yagisawa H Suh PG Ryu SH Phosphatidylinositol (3,4,5)-trisphosphate specifically interacts with the phox homology domain of phospholipase D1 and stimulates its activity J Cell Sci 2005 118 4405 4413 10.1242/jcs.02564 16179605

[B23] Zhan Y Virbasius JV Song X Pomerleau DP Zhou GW The p40phox and p47phox PX domains of NADPH oxidase target cell membranes via direct and indirect recruitment by phosphoinositides J Biol Chem 2002 277 4512 4518 10.1074/jbc.M109520200 11729195

[B24] Streit WJ Microglia as neuroprotective, immunocompetent cells of the CNS Glia 2002 40 133 139 10.1002/glia.10154 12379901

[B25] Town T Nikolic V Tan J The microglial "activation" continuum: from innate to adaptive responses J Neuroinflammation 2005 2 24 10.1186/1742-2094-2-24 16259628 PMC1298325

[B26] Lee SC Liu W Dickson DW Brosnan CF Berman JW Cytokine production by human fetal microglia and astrocytes. Differential induction by lipopolysaccharide and IL-1 beta J Immunol 1993 150 2659 2667 8454848

[B27] Block ML Zecca L Hong JS Microglia-mediated neurotoxicity: uncovering the molecular mechanisms Nat Rev Neurosci 2007 8 57 69 10.1038/nrn2038 17180163

[B28] El Khoury J Hickman SE Thomas CA Cao L Silverstein SC Loike JD Scavenger receptor-mediated adhesion of microglia to beta-amyloid fibrils Nature 1996 382 716 719 10.1038/382716a0 8751442

[B29] Husemann J Loike JD Anankov R Febbraio M Silverstein SC Scavenger receptors in neurobiology and neuropathology: their role on microglia and other cells of the nervous system Glia 2002 40 195 205 10.1002/glia.10148 12379907

[B30] Paresce DM Ghosh RN Maxfield FR Microglial cells internalize aggregates of the Alzheimer's disease amyloid beta-protein via a scavenger receptor Neuron 1996 17 553 565 10.1016/S0896-6273(00)80187-7 8816718

[B31] Mitrasinovic OM Murphy GM Jr Accelerated phagocytosis of amyloid-beta by mouse and human microglia overexpressing the macrophage colony-stimulating factor receptor J Biol Chem 2002 277 29889 29896 10.1074/jbc.M200868200 12032144

[B32] Le Y Gong W Tiffany HL Tumanov A Nedospasov S Shen W Dunlop NM Gao JL Murphy PM Oppenheim JJ Wang JM Amyloid (beta)42 activates a G-protein-coupled chemoattractant receptor, FPR-like-1 J Neurosci 2001 21 RC123 11160457 10.1523/JNEUROSCI.21-02-j0003.2001 PMC6763825

[B33] Yan SD Chen X Fu J Chen M Zhu H Roher A Slattery T Zhao L Nagashima M Morser J RAGE and amyloid-beta peptide neurotoxicity in Alzheimer's disease Nature 1996 382 685 691 10.1038/382685a0 8751438

[B34] Bamberger ME Harris ME McDonald DR Husemann J Landreth GE A cell surface receptor complex for fibrillar beta-amyloid mediates microglial activation J Neurosci 2003 23 2665 2674 12684452 10.1523/JNEUROSCI.23-07-02665.2003 PMC6742111

[B35] Bamberger ME Landreth GE Microglial interaction with beta-amyloid: implications for the pathogenesis of Alzheimer's disease Microsc Res Tech 2001 54 59 70 10.1002/jemt.1121 11455613

[B36] Koenigsknecht J Landreth G Microglial phagocytosis of fibrillar beta-amyloid through a beta1 integrin-dependent mechanism J Neurosci 2004 24 9838 9846 10.1523/JNEUROSCI.2557-04.2004 15525768 PMC6730228

[B37] Wilkinson B Koenigsknecht-Talboo J Grommes C Lee CY Landreth G Fibrillar beta-amyloid-stimulated intracellular signaling cascades require Vav for induction of respiratory burst and phagocytosis in monocytes and microglia J Biol Chem 2006 281 20842 20850 10.1074/jbc.M600627200 16728400

[B38] Gahmberg CG Tolvanen M Kotovuori P Leukocyte adhesion--structure and function of human leukocyte beta2-integrins and their cellular ligands Eur J Biochem 1997 245 215 232 10.1111/j.1432-1033.1997.00215.x 9151947

[B39] Plow EF Zhang L A MAC-1 attack: integrin functions directly challenged in knockout mice J Clin Invest 1997 99 1145 1146 10.1172/JCI119267 9077518 PMC507924

[B40] Brachmann SM Yballe CM Innocenti M Deane JA Fruman DA Thomas SM Cantley LC Role of phosphoinositide 3-kinase regulatory isoforms in development and actin rearrangement Mol Cell Biol 2005 25 2593 2606 10.1128/MCB.25.7.2593-2606.2005 15767666 PMC1061637

[B41] Katso R Okkenhaug K Ahmadi K White S Timms J Waterfield MD Cellular function of phosphoinositide 3-kinases: implications for development, homeostasis, and cancer Annu Rev Cell Dev Biol 2001 17 615 675 10.1146/annurev.cellbio.17.1.615 11687500

[B42] Vanhaesebroeck B Waterfield MD Signaling by distinct classes of phosphoinositide 3-kinases Exp Cell Res 1999 253 239 254 10.1006/excr.1999.4701 10579926

[B43] Deane JA Fruman DA Phosphoinositide 3-kinase: diverse roles in immune cell activation Annu Rev Immunol 2004 22 563 598 10.1146/annurev.immunol.22.012703.104721 15032589

[B44] Cantrell DA Phosphoinositide 3-kinase signalling pathways J Cell Sci 2001 114 1439 1445 11282020 10.1242/jcs.114.8.1439

[B45] Welch HC Coadwell WJ Stephens LR Hawkins PT Phosphoinositide 3-kinase-dependent activation of Rac FEBS Lett 2003 546 93 97 10.1016/S0014-5793(03)00454-X 12829242

[B46] Perisic O Wilson MI Karathanassis D Bravo J Pacold ME Ellson CD Hawkins PT Stephens L Williams RL The role of phosphoinositides and phosphorylation in regulation of NADPH oxidase Adv Enzyme Regul 2004 44 279 298 15581496 10.1016/j.advenzreg.2003.11.003

[B47] Cross AR Segal AW The NADPH oxidase of professional phagocytes--prototype of the NOX electron transport chain systems Biochim Biophys Acta 2004 1657 1 22 15238208 10.1016/j.bbabio.2004.03.008 PMC2636547

[B48] Sheppard FR Kelher MR Moore EE McLaughlin NJ Banerjee A Silliman CC Structural organization of the neutrophil NADPH oxidase: phosphorylation and translocation during priming and activation J Leukoc Biol 2005 78 1025 1042 10.1189/jlb.0804442 16204621

[B49] Reed-Geaghan EG Savage JC Hise AG Landreth GE CD14 and toll-like receptors 2 and 4 are required for fibrillar A{beta}-stimulated microglial activation J Neurosci 2009 29 11982 11992 10.1523/JNEUROSCI.3158-09.2009 19776284 PMC2778845

[B50] Ehlers MR CR3: a general purpose adhesion-recognition receptor essential for innate immunity Microbes Infect 2000 2 289 294 10.1016/S1286-4579(00)00299-9 10758405

[B51] Newham P Humphries MJ Integrin adhesion receptors: structure, function and implications for biomedicine Mol Med Today 1996 2 304 313 10.1016/1357-4310(96)10021-6 8796911

[B52] Strohmeyer R Ramirez M Cole GJ Mueller K Rogers J Association of factor H of the alternative pathway of complement with agrin and complement receptor 3 in the Alzheimer's disease brain J Neuroimmunol 2002 131 135 146 10.1016/S0165-5728(02)00272-2 12458045

